# Simulation-Based Design and Optimization of Rectangular Micro-Cantilever-Based Aerosols Mass Sensor

**DOI:** 10.3390/s20030626

**Published:** 2020-01-22

**Authors:** Feng Xu, Yuliang Wei, Shiyuan Bian, Huanqin Wang, Da-Ren Chen, Deyi Kong

**Affiliations:** 1 State Key Laboratory of Transducer Technology, Key Laboratory of Biomimetic Sensing and Advanced Robot Technology of Anhui Province, Institute of Intelligent Machines, Chinese Academy of Sciences, Hefei 230031, China; xf1992@mail.ustc.edu.cn (F.X.); wyl1010@mail.ustc.edu.cn (Y.W.); sybian@iim.ac.cn (S.B.); 2 Science Island branch of Graduate School, University of Science and Technology of China, Hefei 230009, China; 3 Particle Laboratory, Department of Mechanical and Nuclear Engineering, Virginia Commonwealth University, 401 West Main Street, Richmond, VA 23220, USA

**Keywords:** micro-cantilever, aerosol mass sensor, length to width ratio, slot, edge serration, fluid-structure interaction

## Abstract

Micro-Cantilever (MCL) is a thin film structure that is applied for aerosol particle mass sensing. Several modifications to the rectangular MCL (length-to-width ratio, slots at the anchor, serrations at its side edges) are made to deduce the role and influence of the shape of rectangular MCL-based aerosol mass sensors and reduce gas damping. A finite element fluid-structure interaction model was used to investigate the performance of MCL. It is found that (I) the mass sensitivity and quality factor decline with the increasing of length-to-width ratio which alters the resonant frequency of the MCL. The optimum conditions, including the length-to-width ratio (*σ_lw_* = 5) and resonant frequency (*f*_0_ = 540.7 kHz) of the MCL, are obtained with the constant surface area (*S* = 45,000 μm^2^) in the frequency domain ranging from 0 to 600 kHz. (II) The slots can enhance the read-out signal and bring a small Q factor drop. (III) The edge serrations on MCL significantly reduce the gas damping. The results provide a reference for the design of aerosol mass sensor, which makes it possible to develop aerosol mass sensor with high frequency, sensitivity, and quality.

## 1. Introduction

Particulate matter (PM) in air, considered to be one of the air pollutants, often results in the reduction of visibility and agricultural crop yield due to light dispersion and dissipation effect. Due to their small sizes and high surface-to-volume ratio, fine and ultrafine PM can also penetrate deeply into human bronchi and alveoli, causing/aggravating respiratory diseases Researchers have paid much attention to develop instruments and sensors for measuring aerosol mass concentration (due to the present air quality standards). The aerosol mass sensing method has evolved through two generation according to the volume of instruments [1].

Instruments in the early generation are designed for scientific studies. They are typically bulky in the package, such as standard gravimetric measurement, Beta Gauge [2], Tapered Element Oscillating Microbalance (TEOM) [3,4,5], Berner Low Pressure Impactor (ELPI) [6], and Nephelometric [7]. While they are widely applied in environmental monitoring, they remain expensive in price, labor-intensive for maintenance, and bulky in packaging. For monitoring the PM exposure at the individual level and for distributed measurement tasks, the aerosol mass sensors need to be portable and easily accessible. Therefore, the latest generation of aerosol mass sensing was proposed for the monitoring described above. Based on the operational principle, PM sensors can be categorized into acoustic wave and resonant mass sensors, such as the Micro-Electro-Mechanical Systems (MEMS) or Nano-Electro-Mechanical Systems (NEMS). MEMS/NEMS resonators have become a hot topic due to their superior sensitivity, Q-factor, resonant frequency, and tunable surface area [8,9]. The MEMS/NEMS mass sensors show a better sensitivity than those typical acoustic wave mass sensors. In addition, MEMS/NEMS mass sensors are compatible with Complementary Metal Oxide Semiconductor (CMOS) sensors on a silicon chip. They could eventually be the personalized, low-cost, low power consumption aerosol mass-concentration sensors.

MEMS/NEMS resonant mass sensors can be classified into two categories: bulk mode-based and cantilever-based. In recent decades, researchers have used NEMS resonators to measure spores and virus mass, the sensitivity reached the ‘fg’ level [10,11,12]. NEMS mass sensors even show the potential for a single nanoparticle detection because of its small dimension, low weight, and ultra-high frequency. It is known that the mass sensitivity increases with the decrease of resonator dimensions. However, the detection limit, cycle time, sensitivity, Q factor and other performance parameters of resonator are all affected by the effective area. Hence, the effective area for sensing aerosol mass in the sensors should be considered when measuring micrometer-sized airborne particles.

Micro-Cantilever (MCL) is a thin film structure realized by surface micromachining that was applied for aerosol particle mass sensing due to its high sensitivity, small dimension, high Q-factor, and low power consumption. Cantilever-based MEMS sensors (MCL, Micro-cantilever) consist of a micron beam of one-end rigidly fixed and the other end suspended and bendable. There are two working modes for MCL-based mass sensors, i.e., static mode and dynamic mode. In the dynamic mode, the deposited mass of aerosol particles, resulted in additional mass on the cantilever, is measured by the shift of MCL resonant frequency. Researchers have proposed piezoresistive silicon resonant MEMS cantilever-based sensors for the detection of airborne ultrafine particles (i.e., CANTOR). The CANTOR used electrostatic precipitation as the aerosol sampling method. However, its sampling efficiency requires to be improved (i.e., 1.32%) and its sampling time is relatively long (i.e., 15 min). The latest sensitivity of the CANTOR was 5 μg/m^3^ [13]. In another MCL, a piezoelectric stack actuator and a piezoresistive strain gauge are involved in the sensor system [14]. The mass sensitivity in air is 10 Hz/ng. The influence of ambient temperature, relative humidity, and pressure on the sensor have also been investigated. As a result, the sensor exhibits low cross-sensitivity of <3% due to environmental effects, sensor geometry, detection method, and NP collection system have to be optimized for the measuring the mass of the target substance normally in the atmosphere. To prolong MCL’s operating life, two rectangular silicon MCLs C*_A_* (2750 μm × 100 μm × 50 μm) and C*_B_* (5000 μm × 200 μm × 40 μm) were characterized in resonance [15], a sacrificial photoresist layer is used to avoid direct contact between MCL body and particles. The other two rectangular silicon MCLs Cantor-1 (2750 μm × 100 μm × 50 μm) and Cantor-2 (1000 μm × 170 μm × 19 μm) were described with respect to its behavior under carbon aerosol exposition [16]. The settling times were drastically reduced enabling a time resolution by the redesign of sampling head. The working frequency of the Cantor-1 is 9.3–9.4 kHz, and the limit of detection is 25 μg/m^3^, Q-factor is about 1665. The working frequency of the Cantor-2 is 201.8–202.1 kHz, and the limit of detection is 5 μg/m^3^, Q-factor is about 4702. The shape, mass (or volume) of the MCL affect its detection performance. There are also unconventional uses of micro cantilever [17], the feasibility of detecting gas composition ratio by using different damping of MCL in different gases was evaluated by physical analytical modeling. However, the effect of MCL-based structure in the performance of aerosol mass concentration detection has not been extensively investigated.

MCLs can be profoundly influenced by the air that surrounds them, the Q-factor of an MCL is one of the main dynamic characteristics that are very sensitive to the surrounding fluid properties. Thus, promising to have great potential for reducing the damping of the MCL. Instead of time-consuming and costly MEMS fabrication process, the simulation-based method will minimize the cost and time in the design and optimization. At a small scale, resonators [18,19], gyroscopes [20], accelerometers [21,22], and actuators [23] must consider the physical phenomena involved in their operation in order to design and optimize the structure of MCL. A general theoretical model for the frequency response of an MCL executing torsional vibration in a fluid of arbitrary viscosity and density was presented [24]. However, the principal assumptions must be satisfied that the MCL length greatly exceeds its width, the amplitude of vibration is small, and the fluid is incompressible in nature. Another model of the resonant frequencies of rectangular cantilever beams immersed in fluid was presented [25], which rigorously valid for arbitrary mode number and for cases where the fluid maybe considered to be in viscid in nature. This model is directly applicable to cantilever beams of macroscopic size. The hydrodynamic loading of elastic MCLs vibrating in viscous fluids is analyzed computationally using a finite element fluid-structure interaction model [26]. The results indicate that MCL damping arises from localized fluid shear near the edges of the MCL. The MCL-based aerosols mass sensor is operated in air, which is easier to compress than liquid-phase fluids. In measurement of atmospheric or compressible fluid, as the mode number of MCL increases and passes a “coincidence point”, the Q factor will finally start to decrease [27,28]. However, the result is different in the incompressible fluid [29,30,31,32]. Since the compression of the gas leads to additional acoustic damping, it was previously assumed to be negligible in the incompressible fluid [33].

In the present study, the solid MCLs and surrounding air are modeled respectively using shell elements and Thermoviscous Acoustics and Pressure Acoustic. The effects of length-to-width ratios (*σ_lw_*) on the aerosol mass sensor with a constant thickness is critically investigated. Furthermore, it is the first time to add edge serration to MCL. Combined with a slotted structure, the read-out signal strength can be enhanced. Meanwhile, the edge serration on the MCL-based aerosols sensors can reduce the damping in air fluid. Thus, it will greatly reduce the damping limitation of the MCL in fluid detection under high frequency mode.

## 2. Materials and Methods

### 2.1. Principle of the MCL-Based Mass Sensors

**Figure 1:**
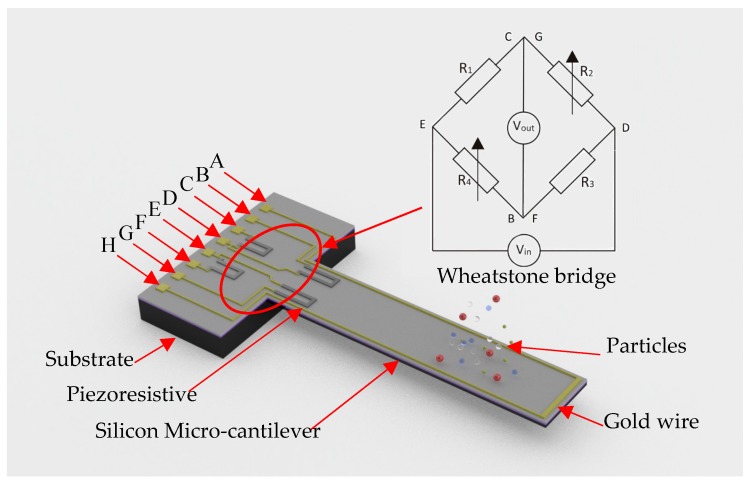
3D Visualization of the MCL-based mass sensor. A: actuate voltage (+); B and F: output voltage (+); C and G: output voltage (−); E: input voltage (+); D: input voltage (−).

The actuate methods of MCL are piezoelectric [34,35,36], electric [37,38], flexoelectric [39], atomic force [40], magnetic [41], ultrasound radiation force [42], and laser irradiation [43]. The methods of reading out the output signal of MCL include optics [44,45], piezoresistance [46,47], piezoelectricity [48], capacitive [49], and tunneling [50]. As an example, there is a magnetic-actuating MCL, be used as a sensing element as shown in Figure 1, which can be made of Silicon-On-Insulator (SOI). The MCL placed in the magnetic field can be actuated, when an alternating current is applied to the A and H pins. Making piezoresistance on the surface of MCL is a typical MEMS technology. For example, we can make it with Silicon-On-Insulator (SOI), which’s thickness of the top layer is 5 μm, and the resistivity is 1–10 Ω·cm. Piezoresistance on the MCL obtained by boron (B)-doping of the n-type silicon. The injection energy is 60 keV, the dose is 8 × 10^14^/cm^3^, and the photoresist is used as mask. After the photoresist is removed, it is annealed at 950 °C in nitrogen atmosphere for 30 min. A piezoresistance of about 380 Ω is obtained. The output signal of the MCL is read through the output pins of the Diagonal Wheatstone Bridge (B/F and C/G). An input DC voltage, *V*_in_, is across the E and D terminals and the output in the form of voltage, *V*_out_, is measured across C/G and B/F terminals. In dynamic mode, the deflects of the vibration resulting in a change of resistance of the piezoresistor placed on MCL (R_2_&R_4_). The output voltage can be expressed as:

(1) Vout = (R2R1+R2−R3R3+R4)Vin.

Accordingly, the frequency of the MCL can be converted into the frequency of the *V*_out._ The mass of particles loaded on the surface of the MCL can be calculated by measuring the resonant frequency shift of the MCL. The resonant behavior of MCL can be described by Hooke’s law. For rectangular MCL, its effective stiffness/spring constant $k_{\mathrm{eff}}$ can be obtained from

(2) keff = Eb34L3,

the effective mass ($m_{\mathrm{eff}}$) is:

(3) meff = 3ρLWb(1.875)4 ≈ ρLWb4,

and the resonant frequency of the MCL (*f*_0_) is:

(4) f0 = 12πkeffmeff,

where E, b, W, and L are the elastic modulus, thickness, width and length of MCL respectively [51]. The π is the ratio of a circle’s circumference. The ρ is the density of the MCL. When the MCL is loaded with particles, the resonant frequency of the MCL becomes *f*_1_ The mass changes (Δm) can calculated from:

(5) Δm = keff4π2(1f12−1f02).

The airborne particulate mass concentration (*X*) is:

(6) X = ΔmηTlQl,

where the η is the adsorption efficiency of airborne particles, *T*_1_ and *Q*_1_ are the sampling/deposition time of the airborne particles and flow rate during sampling, respectively. Therefore, the mass concentration of particles can be obtained by detecting the resonant frequency shift of MCL.

### 2.2. Theory

The resonant frequency of MCL in vacuum can be calculated by Equation (3). The resonant frequency of MCL in air will be affected by air fluid. Each resonant frequency of MCL in vacuum can be calculated by the formula [51]:

(7) $$f_{n}\;=\;\frac{\beta_{n}^{2}}{{2\pi L}^{2}}\sqrt{\frac{{Eb}^{2}}{12\rho }},$$

where the $f_{n}$ is resonant frequency corresponding to different modes, $\beta_{n}$ is coefficient related to the eigenvalue of each harmonic, $\beta_{n}\;=\;\pi (n-0.5)$, n = 1, 2, …, n. When the MCL vibrates in the air, the resonant frequency ($f_{n\_\mathrm{Air}}$) is [24,52]:

(8) $$f_{n\_\mathrm{Air}}{\;=\;f}_{n}{\left(1+\frac{{\pi \rho }_{t}W}{4\rho b}\right)}^{-1/2}\;=\;\frac{\beta_{n}^{2}}{{2\pi L}^{2}}\sqrt{\frac{{Eb}^{2}}{12\rho }}{\left(1+\frac{{\pi \rho }_{t}W}{4\rho b}\right)}^{-1/2}.$$

The Q-factor determine the performance of the MCL-based aerosols mass sensor which influenced by fluid damping and the shapes. The Q-factor refers to the resolution for the MCL-based aerosols mass sensor. As we known, it can be defined as the ratio of the total energy stored in the vibration system ($E_{m}$) to the energy lost ($E_{c}$) in the vibration period. The Q-factor can be characterized as follows:

(9) $$Q\;=\;2\pi (\frac{E_{m}}{E_{c}}).$$

Thermal and viscous damping of the MCL also needs to be considered. It is necessary to include thermal conduction effects and viscous losses explicitly in the governing equation. The governing equations for the fluctuation (wave equation or Helmholtz equation) are derived by perturbing or linearizing the Navier–Stokes equations, momentum equation, continuity equation and energy equation [53]. This is achieved by solving the thermoviscous acoustics equations in the Thermoviscous Acoustics interfaces in the COMSOL Acoustics Module.

The continuity equation for Thermoviscous Acoustics:

(10) $${iw\rho }_{t}+\;\nabla \cdot {(\rho }_{0}u_{t})\;=\;0.$$

The conservation equations for momentum and energy for acoustic perturbation:

(11) $${iw\rho }_{0}u\;=\;\nabla \cdot (-p_{t}I\;+\;µ(\nabla u_{t}\;+(\nabla u_{t}{)}^{T}{\;)\;+\;(µ}_{B}-\frac{2}{3}µ)(\nabla \cdot u_{t})I),$$

(12) $$iw\left(\rho_{0}C_{p}T_{t}\;-{\;T}_{0}\alpha_{0}p_{t}\right)\;=\;-\nabla \cdot (-\lambda \nabla T_{t}{)\;+\;Q}_{h}.$$

And in the fluid domains, the pressure distribution is governed by the wave equation:

(13) $$\nabla \cdot \left(-\frac{1}{\rho_{0}}\left(\nabla p_{t}-q_{d}\right)\right)-\;\frac{\;w^{2}p_{t}}{c^{2}\rho_{0}}{\;=\;Q}_{m}.$$

The dependent variables take the form:

(14) $$p_{t}\;=\;p_{0}\;+\;p^{\prime }e^{iwt},\;u_{t}\;=\;u_{0}\;+\;u^{\prime }e^{iwt},\;T_{t}\;=\;T_{0}\;+\;T^{\prime }e^{iwt},$$

where *p*, *u*, and *T* are the pressure, velocity and temperature of air around MCL, respectively, and w is the angular frequency. Where $\alpha_{0}$ is the coefficient of thermal expansion (isobaric), $Q_{h}\;$ and $Q_{m}\;$ are a possible heat source and the monopole source, respectively, and the $q_{d}$ is the dipole source. Variables with sign (‘) are acoustic variables, and variables with subscript (0) represent background mean flow quantities.

### 2.3. Design

To investigate the effects of $\sigma_{lw}$, slots, and edge serrations on the detection performance, the MCLs with different $\sigma_{lw}$ and special structure are set up. In the simulation, modeled using shell elements, the MCL can be simplified to a spring structure with anchor fixed. It should be noted that the particles were considered to load on the upper surface of the MCL approximately uniformly. The mechanical properties of silicon material embedded in COMSOL were compiled in Table 1, they come from the default properties of silicon dynamic analysis in the MEMS module of COMSOL.

#### 2.3.1. The Shapes of MCLs

**Figure 2:**
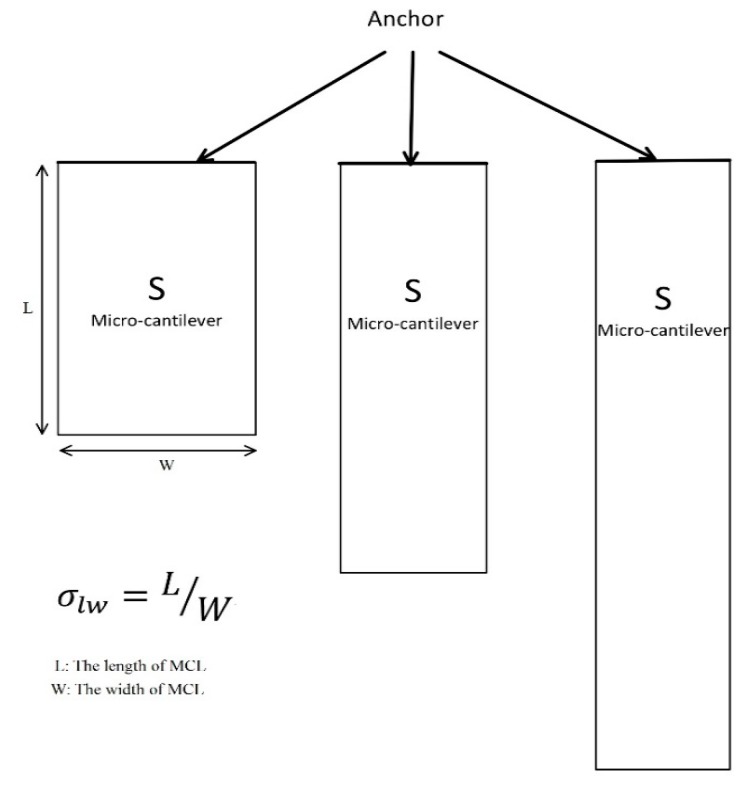
Top view of the MCLs geometry with different length to width ratios ($\sigma_{lw}=1,\;2,\;3,\;4,\;5,\;6,\;7,\;8,\;9$).

Different shapes affect the detection performance of micro-cantilever. For rectangular shell structure, the $\sigma_{lw}$ is mainly considered. Different length to width ratios ($\sigma_{lw}\;=\;1,\;2,\;3,\;4,\;5,\;6,\;7,\;8,\;9$) were set to investigate the effects of different shapes as shown in Figure 2. To eliminate the influence of the effective mass of the MCL, the thickness of the MCLs are set to 5 μm and the effective area (tunable surface area, S) is 4.5 × 10^4^ μm^2^.

#### 2.3.2. The Slots of MCLs

The rate of resistance change of the piezoresistive resistance can be described as:

(15) $$\frac{{dR}_{p}\;}{R_{p}}\;=\;\left(\pi E\;+\;1\;+\;2µ\right)\varepsilon ,$$

where the *E* is the elastic modulus, the µ is Poisson’s ratio, and the  ε is strain.

**Figure 3:**
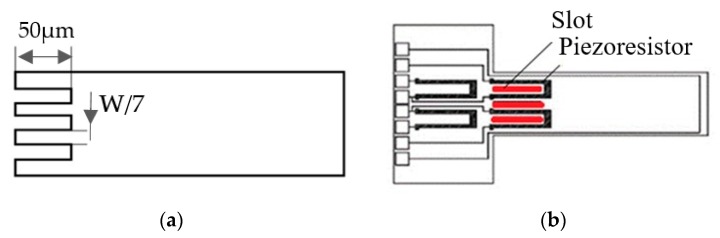
Top view of the slotted MCL-based mass sensor geometry. (**a**) Simplified outline of slotted MCL; (**b**) Geometry of slotted MCL with Wheatstone bridge.

High rate of resistance change is beneficial to improve the signal intensity of the detection system, especially in MEMS sensors. In dynamic mode, the strain ε is positively correlated with the deflection of MCL in piezoresistive attachment area (0 μm < L < 50 μm). In addition, the deflection is also positively correlated with other signal readout methods. Hence, the read-out signal strength increases with the deflection. As shown in Figure 3a, three slots are arranged at the anchor end of the MCL. The length of the slots is 50 μm, the width of the slots is W/7 for each shape. The deflection, mass sensitivity, power dissipation, and Q-factor of the MCL will be investigated.

#### 2.3.3. The Edge Serrations of MCLs

**Figure 4:**
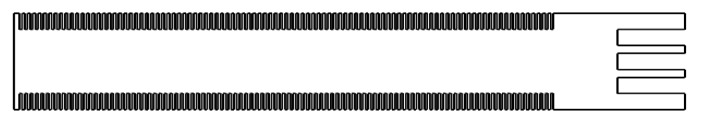
Geometry of slotted MCL with edge serrations.

The edge serrations structure can change the flow state of the fluid to bring about some improvements. For example, the leading-edge serrations on the owl’s wings allow him to achieve remarkably low noise gliding and flapping flights [54,55]. It is assumed that the edge serrations also reduce the disturbance to the fluid in the micro length scale. The effect of edge serrations on MCLs were investigated by simulation. As shown in Figure 4, edge serrations of 15 μm by 2 μm are added to the both free edge connected to the anchor end of MCL.

#### 2.3.4. Material Property of Air

According to Newton’s law of viscosity, the dynamic viscosity (μ) of air is significantly correlated with temperature, but almost independent of pressure. When the temperature *T* is less than 2000 K, the dynamic viscosity can be calculated by Sutherland formula [56]:

(16) $$\frac{\mu_{B}}{\mu_{0}}\;=\;\;\frac{T_{0}\;+\;B}{T\;+\;B}{\left(\frac{T}{T_{0}}\right)}^{3/2},$$

where $\mu_{B}$ is the reference dynamic viscosity corresponding to the reference temperature ($T_{0}$). Where *B* is a constant related to the type of gas (*B* of air is 110.4 K). We need to simulate thermal and viscous damping, so we need to consider the influence of temperature on the heat capacity and thermal conductivity of air. The heat capacity ($C_{P}$) of air was described by empirical formulas:

(17) $$C_{P}{\;=\;a\;+\;bT}^{1}{\;+\;cT}^{2}{\;+\;dT}^{3}{\;+\;eT}^{4}\;+\;\ldots \;(200\;<\;T\;<\;1600\;K),$$

where a, b, c, d, and e are empirical constants: a = 1047.63657 J/(kg∙K); b = −0.372589265 J/(kg∙K^3^); c = 9.45304214 × 10^−4^ J/(kg∙K^3^); d = −6.02409443 × 10^−7^ J/(kg∙K^4^); e = 1.2858961 × 10^−10^ J/(kg∙K^5^). Similarly, the thermal conductivity (λ) of air can be obtained by empirical formula:

(18) $${\lambda \;=\;a\;+\;bT}^{1}{\;+\;cT}^{2}{\;+\;dT}^{3}{\;+\;eT}^{4}\;+\;\ldots \;(200\;<\;T\;<\;1600\;K),$$

where a, b, c, d, and e are empirical constants: a = −0.00227583562 W/(kg∙K); b = 1.15480022 × 10^−4^ W/(kg∙K^2^); c = −7.90252856 × 10^−8^ W/(kg∙K^3^); d = 4.11702505 × 10^−11^ W/(kg∙K^4^); e = −7.43864331 × 10^−15^ W/(kg∙K^4^).The relationship between air density, temperature and pressure can be described by the Clapeyron equation [57]:

(19) $$\rho_{t}\;=\;\frac{kP}{R_{c}T},$$

where $\rho_{t}$ is the density of air corresponding to the temperature (T), *P* is pressure. Where *k* and *R* constants related to the type of gas (*k* of air is 0.02897, $R_{c}$ of air is about 8314 Pa·L/mol·K). In the flow field where the temperature *T* is not constant, the sound velocity at each point is different. The expressions of sound velocity (*u*) and temperature are as follows:

(20) $$u\;=\;\sqrt{\lambda_{0}RT},$$

where $\lambda_{0}$ and *R* are the specific heat ratio and the universal gas constant ($\lambda_{0}$ of air is 1.4, *R* of air is 287.14 J ${\cdot \mathrm{kg}}^{\;-\;1}\cdot K^{\;-\;1}$).

### 2.4. Modeling and Validation

**Figure 5:**
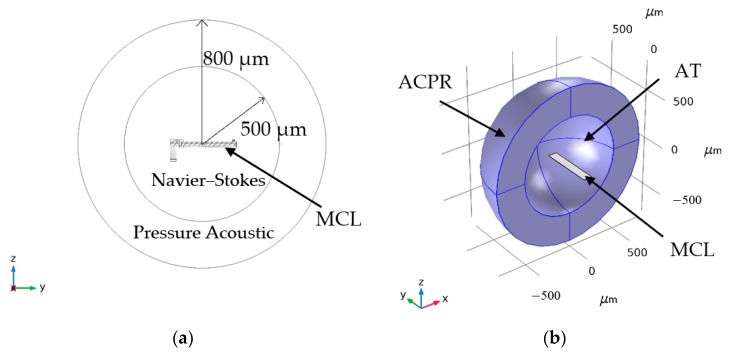
Geometry of air around the MCLs. (**a**) A cross-sectional schematic of the 3D finite element model; (**b**) A 3D cut image of the finite element model.

The model is established and calculated by using the COMSOL Multiphysics 5.3a. A sketch of MCL modeled are shown in Figure 2, Figure 3 and Figure 4. The solid MCLs are modeled using shell elements and the surrounding air is modeled using Thermoviscous Acoustics and Pressure Acoustic [58]. When vibration waves propagate in a fluid bounded by the surface of MCL, so-called viscous and thermal boundary layers are created at the MCL surfaces. The temperature of the atmosphere is 20 °C, and the pressure is standard atmospheric pressure (1 atm). There is a typical spherical geometry is taken as an approximation of the actual geometry [26,53]. The MCL is submerged in a 3D enclosure of Navier–Stokes elements of an incompressible viscous fluid which is set as the “Thermoviscous Acoustics, frequency domain” boundary (TA). These Navier–Stokes fluid elements are then surrounded by Pressure Acoustic 3D fluid elements which is set as the “Pressure Acoustic, frequency domain” boundary (ACPR). For the case of MCL submerged in air of infinite extent and ensure the convergence of the model [26], as shown in Figure 5, the air geometry is plotted as a sphere with a diameter of 800 μm. The central sphere with an inner diameter of 500 μm.

The validation data are used to evaluate the simulation method based on the practical example of these fabricated rectangular MCL for detecting airborne particles. The performance of these fabricated MCLs with different dimensions was reported. We compared the experimental data with the theoretical model simulation data from this work. As the goal was to verify the accuracy of the simulation model, the focus of the validation was on the basic performance of MCL in the air at the micro scale rather than other performance aspects.

## 3. Results and Discussion

### 3.1. Simulation-Model Validation

To verify the accuracy of the simulation model, the model of this work is used to simulate the basic performance of MCLs as listed in Table 2. The data from experimental and simulation are compared. To verify the validity of the method and the obtained results of the theoretical model. As expected, most of the experimental and simulation data have minor differences (<10%). It is noted that there is only relative error more than 20% (24%) in the Q factor of MCL with dimension 2750 μm × 100 μm × 50 μm in first vibration mode. The difference is more obvious in the larger amplitude vibration mode (first vibration mode) of the smaller MCL. These errors may come from the difference between the actual parameters of the measurement environment and the simulated settings. As mentioned above, the Q-factor will finally start to decrease in measurement of atmospheric as the mode number of MCL increases and passes a “coincidence point”. This is also confirmed on the third vibration mode MCL with size 2750 μm × 100 μm × 50 μm. Nonetheless, the relative error between experimental data and simulation data is very small. It is verified that the multi physical field coupling model can precisely simulate the performance of micro-cantilever in detecting airborne particles.

### 3.2. The Effects of MCLs’ Shapes

As we know, the dimension of resonator will directly affect its detection performance. To explore the influence of the length width ratio of the rectangular MCL on the detection performance, it is impossible to compare the MCL with different volume or even with different area. We therefore eliminate the influence factors of volume and effective area in constant volume, and analyze the influence of the shapes of MCLs on the detection of airborne particles. Frequency search range is set to 0–600 kHz.

#### 3.2.1. The Vibration Mode

MCL have different actuating, sampling, and signal read-out methods. It is necessary to refer to the vibration mode to evaluate whether it can perform normally when determining the working frequency and sampling method of the MCL-based aerosols sensors. Too high frequency and too large deflection will lead to the particle separation from the resonator. To investigate the vibration mode, a set of simulations are used to investigate the performance of the MCLs with different shapes.

**Figure 6:**
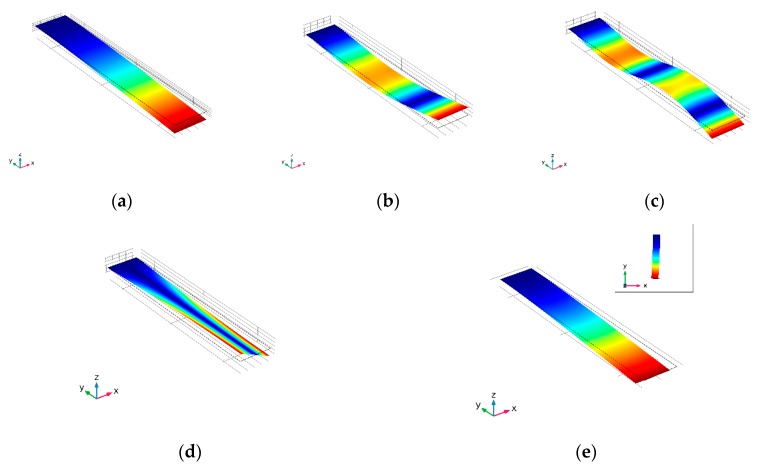
3D Visualization of simulated deflection with the maximum value. (**a**) 3D Visualization of simulated deflection in 1st *z*-Bending mode; (**b**) 3D Visualization of simulated deflection in 2nd *z*-Bending mode; (**c**) 3D Visualization of simulated deflection in 3rd *z*-Bending mode; (**d**) 3D Visualization of simulated deflection in Torsional mode; (**e**) 3D Visualization of simulated deflection in *x*-Bending mode.

As shown in Figure 6, there are three types of vibration modes of MCLs with different shapes. The first vibration mode is the standard bending mode (z-Bending mode), different waveform numbers are the characteristics that distinguish them. The second mode is torsional mode. The former two types of vibration modes can adopt the conventional actuating and sampling methods. The vibration direction of the x-Bending is along the direction of the shell surface. This is bad for sampling, since the transverse vibration will make the particles separate from the effective area easily. The actuating and signal read-out methods applicable to different vibration modes of MCLs are shown in Table 3. The actuating and signal read-out methods in torsional mode are limited. As the example of MCL proposed above, the Lorentz force, which is periodically changing in the magnetic field, is used to actuate the torsional vibration. Therefore, the bending mode of MCL is more general. Besides, the order and frequency position of each vibration mode are different between MCL with different $\sigma_{lw}$, The $\;\sigma_{lw}$ affects the order and resonant frequency of various modes of MCL.

#### 3.2.2. The Detection Performance

To evaluate the detection performance, the mass sensitivity of MCL structure, Q-factor and power dissipation of MCLs structure are simulated. The mass sensitivity is calculated by simulating the frequency shift of the MCL loaded with 1 ng and 2 ng particles, the Q-factor is simulated through frequency-domain sweep, and the power dissipation of one vibration period are calculated by integral power density.

**Figure 7:**
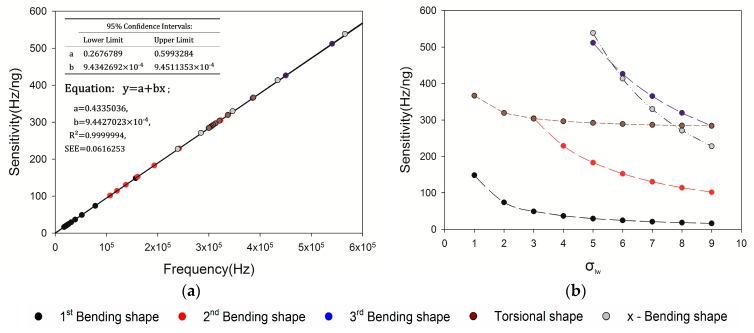
Mass sensitivity of MCLs structure. (**a**) Linear fit of mass sensitivity and resonant frequency of MCLs. (**b**) Sensitivity changes with different $\sigma_{lw}$ of MCLs.

Figure 7a shows the linear fit of mass sensitivity and working frequency of MCLs. Compared to the continuous line, it is evident that the linear relationship between resonant frequency and mass sensitivity. In other words, the influence of $\sigma_{lw}$ of MCLs on the linear relationship between frequency and mass sensitivity is almost negligible. However, it is safe to say that different shapes have their resonant frequencies, which determine the sensitivity. The mass sensitivity is inversely proportional to the $\sigma_{lw}$ of MCLs in each vibration mode as shown in Figure 7b.

**Figure 8:**
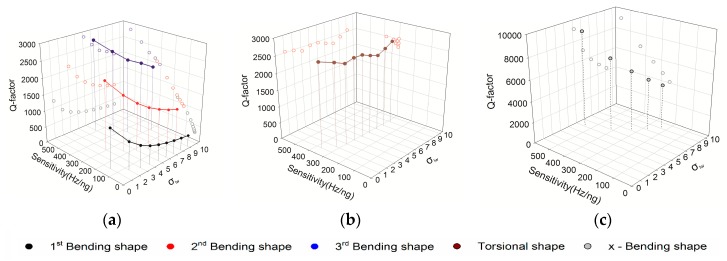
Q-factor variation of MCLs structural. (**a**) Q-factors of MCLs in *z*-Bending vibration mode, (**b**) Q-factors of MCLs in torsional vibration mode, (**c**) Q-factors of MCLs in *x*-Bending vibration mode.

The Q-factors of MCLs in different vibration modes are depicted in Figure 8. The relationship between mass sensitivity, $\sigma_{lw}$, and Q-factor is demonstrated. In the z-Bending vibration mode, there are three separate curves in the figure, which represent the former three vibration modes respectively. There is a noticeable downward trend with the increase of $\sigma_{lw}$. As we can see, the YZ plane, Q-factor is linearly proportional to mass sensitivity. In other words, the Q-factor is proportional to the resonant frequency. Therefore, it is easy to determine the optimal parameters of MCL in the frequency domain. For example, the best $\sigma_{lw}$ of MCL with the same surface area is 5, and the working frequency is 540.7 kHz in the frequency domain of 0–600 kHz. In the torsional vibration mode, the range of Q-factor and sensitivity is narrow. It was clear that the mass sensitivity, $\sigma_{lw}$ and Q-factor of the MCL have poor correlation. It can be inferred that the thickness of the MCL is the parameter that determines the detection performance of the torsional vibration mode. To further evaluate the performance of this vibration mode, we can compare the power dissipation in the air. In the *x*-Bending vibration mode, the noticeable trend is similar to the *z*-Bending vibration mode. In addition, there are higher Q-factor than other modes.

**Figure 9:**
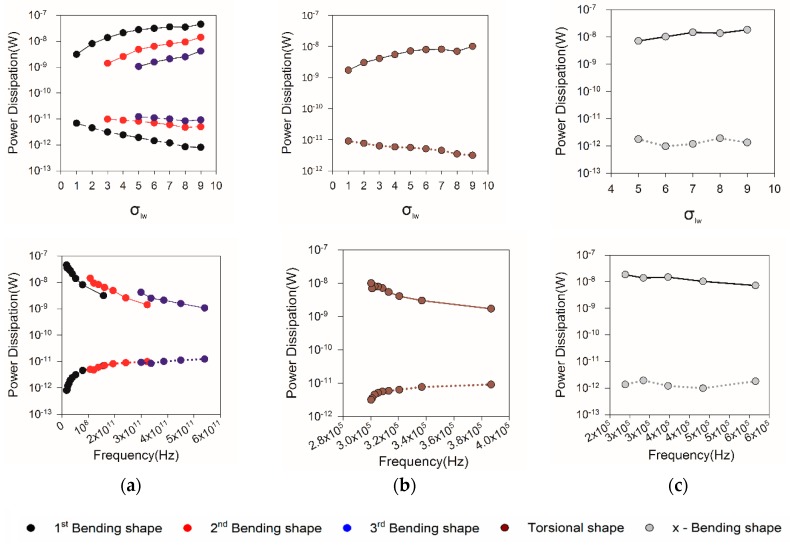
The power dissipation of conventional MCLs. (**a**) The power dissipation of MCLs in *z*-Bending vibration mode, (**b**) The power dissipation of MCLs in torsional vibration mode, (**c**) The power dissipation of MCLs in *x*-Bending vibration mode.

The line graph of the power dissipation corresponding to varying $\sigma_{lw}$ and resonant frequency is shown in Figure 9. The power dissipation is divided into thermal and viscous dissipation, which be denoted by solid line and dotted line. The viscous dissipation increased with increment in $\sigma_{lw}$ of MCLs, while decreased with increment in resonant frequency. In contrast, there is an obviously reversed trend of thermal dissipation. At first, it is crystal clear that the power dissipation primarily comes from viscous dissipation in the search frequency domain. It can be inferred that thermal dissipation will be dominant with the increase of resonant frequency. Graphs confirm the linear relation between the $\sigma_{lw}$ and logarithm of both thermal and viscous dissipation. There is a logarithmic relation between resonant frequency and both thermal and viscous dissipation. In addition, it can be concluded that the thermal dissipation has little correlation with the vibration mode, and its change mainly depends on the value of resonant frequency. However, viscous dissipation is not only related to resonant frequency but also to the vibration mode.

In this section, the shape of conventional MCLs effects on the vibration mode and detection performance is simulated. The $\sigma_{lw}$ of conventional MCLs affect the frequency of various vibration modes and sensitivity. The relationship between Q-factor and $\sigma_{lw}$ is different in each vibration mode. Overall, the Q-factor is inversely proportional to the $\sigma_{lw}$ in the bending modes. The viscous dissipation is increasing and the thermal is decreasing with increment in $\sigma_{lw}$.

### 3.3. Effect of MCL Slots

In this section, a set of simulations are used to investigate the slotted effects on deflection of MCLs.

**Figure 10:**
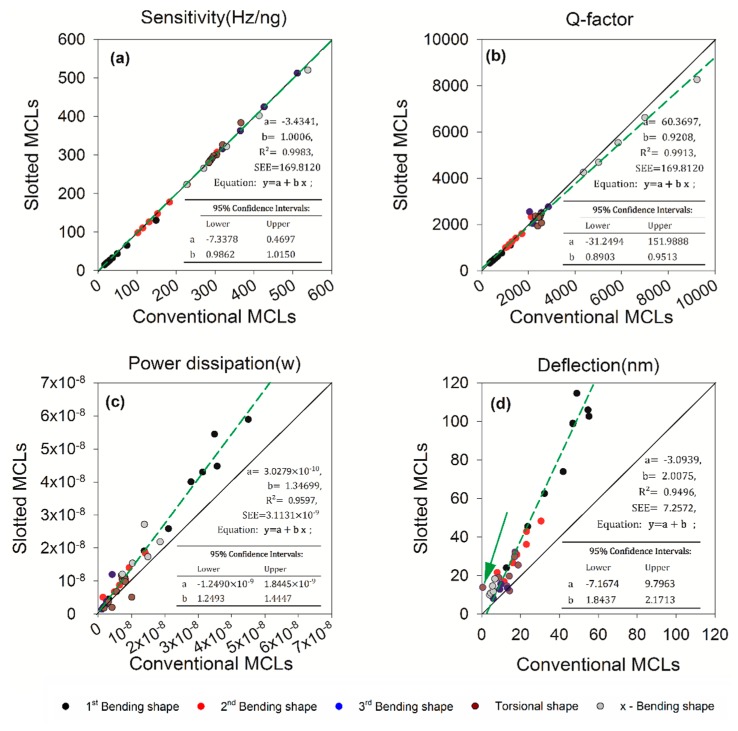
Variation in slotted and conventional MCLs. (**a**) The sensitivity change of structure between slotted and conventional MCLs, (**b**) The Q-factor change between slotted and conventional MCLs, (**c**) The power dissipation between slotted and conventional MCLs, (**d**) The maximum deflection at the piezoresistive resistance area (along MCL length, 50 μm), the green arrow refers to the point ($\sigma_{lw}=$ 1, torsional mode) where the deflection increases is the largest one.

**Figure 11:**
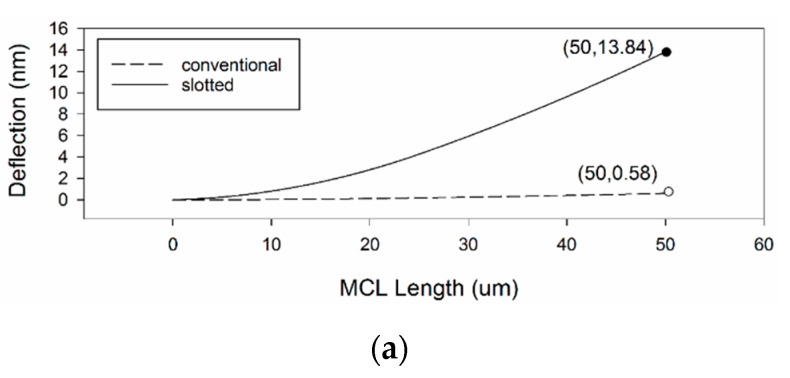

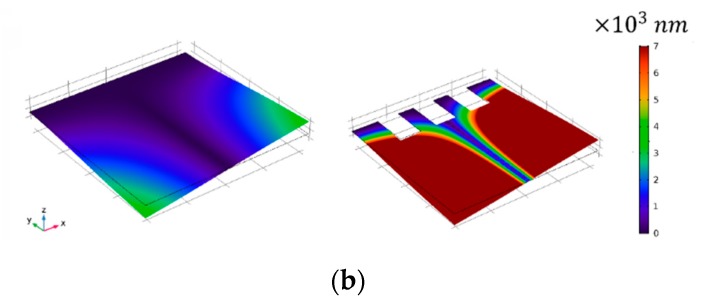
Deflection variation of MCLs along its length. (**a**) Comparison of slotted and conventional MCLs deflection, (**b**) 3D visualization of slotted and conventional MCLs deflection in torsional mode ($\sigma_{lw}=$ 1).

The 1:1 line graph of variation in slotted and conventional MCLs is shown in Figure 10. Compared with the 1:1 line, which is represented as one continuous line. The green dotted line is a linear fit curve. We can see that only the fitting curve of sensitivity is close to the line of 1:1, and others have different degrees of offset. Among them, deflection has the largest deviation. This shows that the deflection of micro cantilevers increases significantly. Besides, there is a significant increase in the power dissipation of the slotted MCLs relative to the conventional MCLs is shown in Figure 10c. This may be due to the fact that the slotted structure increases the deflection of the MCLs. As expected, the maximum deflection at the piezoresistive resistance area with length =50 μm of the slotted MCLs are larger, which is clearly shown in Figure 10d. It is worth noting that the growth rate corresponding to the green arrow in the figure is the largest one. This point corresponds to a torsional mode with $\sigma_{lw}=$ 1. The line graph of variation in deflection of slotted and conventional MCLs with distance to anchor end is shown in Figure 11a. Figure 11b shows 3D visualization of slotted and conventional MCLs deflection in torsional mode. In the torsional mode with $\sigma_{lw}=$ 1, the wider width and the longer anchor end result in an enormous deflection change.

As a conclusion, there is an evident effect of slots on increasing the deflection of MCLs. This will increase the sensitivity of piezoresistive materials. However, it brings the disadvantages of increasing the power dissipation. In general, it is acceptable to enhance the readout weak signals of MCL-based mass sensors at the expense of increased power dissipation in the low frequency range.

### 3.4. Effect of Edge Serration

As we know, viscous and thermal damping are important factors that cannot be ignored when design the MCL-based aerosols mass sensors. We can measure damping amount by the power dissipation caused by damping which is divided into thermal and viscous dissipation. In this section, a set of simulations are used to verify the effect of the micro edge serrations on damping based on the slotted MCL described above ($\sigma_{lw}=6$, in 1st Bending vibration mode and torsional vibration mode).

**Figure 12:**
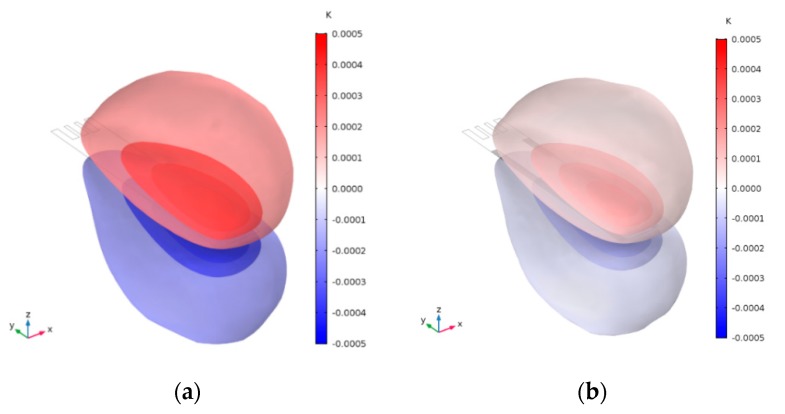

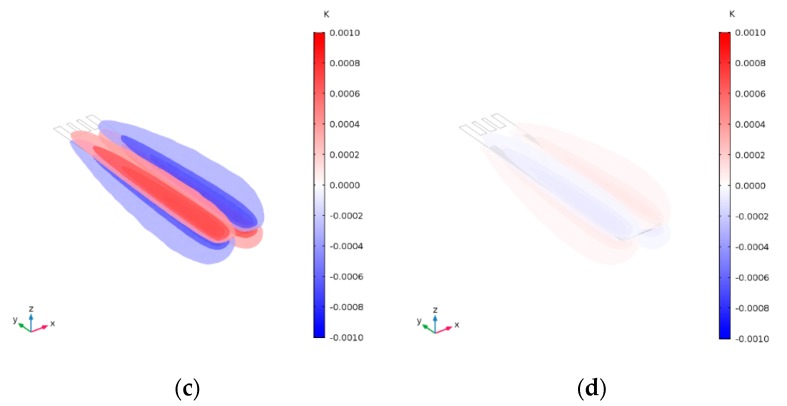
3D Visualization of temperature variation (*K*). (**a**) Temperature variation around the slotted MCL in 1st Bending mode, (**b**) Temperature variation around the slotted MCL with edge serrations in 1st Bending mode, (**c**) Temperature variation around the slotted MCL in torsional mode, (**d**) Temperature variation around the slotted MCL with edge serrations in torsional mode.

The temperature variation of slotted MCL in 1st Bending mode and Torsional mode are shown in Figure 12a,c, respectively. The position of maximum temperature variation is opposite to that of minimum temperature variation. As we know, temperature variation is closely related to pressure variation through equation of state. The temperature gradient near the surface of the MCL under isothermal conditions leads to thermal dissipation. As an obvious contrast, it can be seen that the MCL with edge serrations has a smaller temperature variation as shown in Figure 12b,d. The max temperature variation in slotted MCL and slotted MCL with edge serrations are summarized in Table 4. Lower thermal dissipation is observed in slotted MCL with edge serrations.

**Figure 13:**
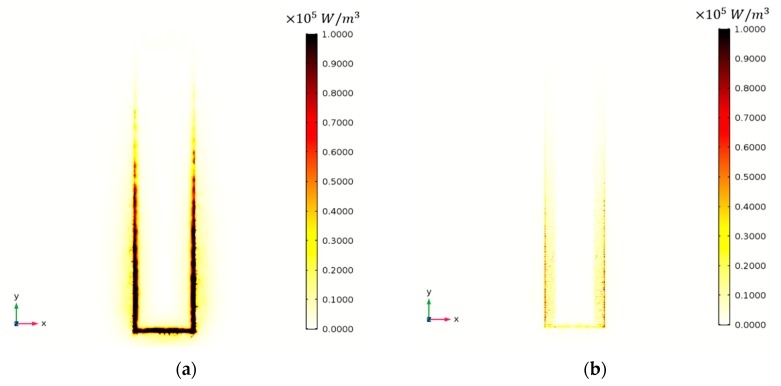

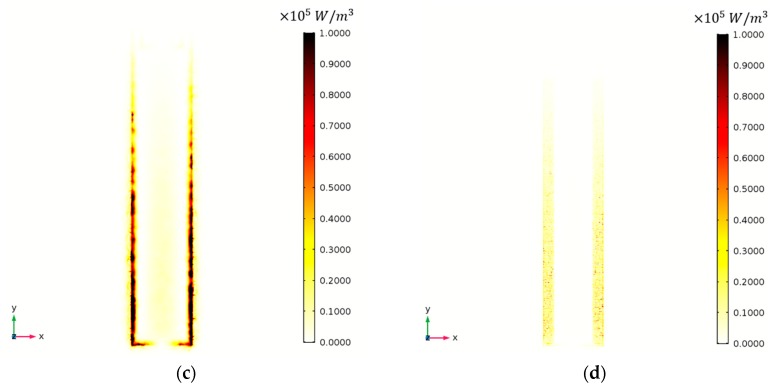
Top view of viscous dissipation density (W/m^3^). (**a**) Viscous dissipation density around the slotted MCL in 1st Bending mode, (**b**) Viscous dissipation density around the slotted MCL with edge serrations in 1st Bending mode, (**c**) Viscous dissipation density around the slotted MCL in torsional mode, (**d**) Viscous dissipation density around the slotted MCL with edge serrations in torsional mode.

There is an obvious difference between slotted MCL and slotted MCL with edge serration in terms of viscous dissipation density, as shown in Figure 13. The maximum viscous dissipation density is concentrated at the edge of the slotted MCL surface. However, it is evenly distributed around the edge serrations of the serrated MCL. The edge serration-based mechanism is in terms of passive control of the airflow, the serrations play a crucial role in reducing flow fluctuations and making changes in the lamina-turbulent transition as well as the turbulence distribution around the edge of MCL. This may because the edge serration on the MCL are equivalent to the air vortex generator that slow down the transition from laminar to turbulent flow.

Table 5 presents the variation in dissipation between slotted MCL and slotted MCL with edge serrations. As concluded above that the edge serration reduces viscous and thermal dissipation, it is found that the viscous damping plays the leading role for the performance of MCLs in constant dimensions working in the frequency domain of 0–600 kHz. In the manufacture of the MCL-based aerosol mass sensors, we should evaluate the influence of different dimensions of edge serration. The key criteria in this part of analysis is not to substantially raise the manufacturing cost.

## 4. Conclusions

A finite element fluid-structure interaction model was used to investigate the designed and optimized the structure of MCL-based aerosol mass sensors. The solid MCLs are modeled using shell elements and the surrounding air is modeled using Thermoviscous Acoustics and Pressure Acoustic. The effects of $\sigma_{lw}$ on the rectangular MCL-based aerosols mass sensor with a constant thickness is investigated in detail. The vibration modes and performance of MCLs in different shapes ($\sigma_{lw}$ = 1, 2, 3, 4, 5, 6, 7, 8, 9) were modeled. It is found that the $\sigma_{lw}$ of conventional MCLs affect the frequency of various vibration modes and sensitivity; the Q-factor is inversely proportional to $\sigma_{lw}$ in the bending modes; the viscous dissipation is increased and the thermal is decreased with increment in $\sigma_{lw}$. Different frequency domain and volume of MCL were varied to optimize the MCL performance. Therefore, the optimal $\sigma_{lw}$ (5) and resonant frequency (540.7 kHz) of the MCL with the constant surface area (45,000 μm^2^) in the frequency domain from 0 kHz to 600 kHz were obtained, the Q factor is 2860.43, the mass sensitivity is 511.78 Hz/ng.

Combined with a slotted structure, the read-out signal can be enhanced. We further found that the MCL slots have obvious effect on increasing the deflection of MCLs, but it introduces the disadvantage of increasing the power dissipation. Therefore, as far as we know, it is the first time researchers added edge serration to the edge of MCL and carried out a qualitative simulation. As expected, the edge serration on the MCL-based aerosols sensors can reduce the damping in fluid. It will greatly reduce the damping limitation of the MCL in fluid detection under high frequency mode.

## Figures and Tables

**Table 1:** Mechanical properties of MCL.

Property	Value	Unit
Density	2329	kg/m^3^
Young’s modulus	170	G Pa
Poisson’s ratio	0.28	1
Thermal expansion coefficient	2.6 × 10^6^	1/K
Thermal conductivity	130	W/(m·K)
Heat capacity at constant pressure	700	J/(kg·K)

**Table 2:** The performance parameters of experimental and simulation.

$\mathrm{Dimension}\;(\mu m^{3})$	Experimental			Simulation		
	Frequency (Hz)	Sensitivity (Hz/ng)	Q	Frequency (Hz)	Sensitivity (Hz/ng)	Q
1250 × 26.5 × 39.3 [13]	~221,502.36	36.5	~1950	216,460.77	35.58	1901.17
1250 × 30 × 25 [14]	~43,900	~10	~1206	46,703.92	10.46	1147.52
1000 × 170 × 19 [16]	~201,950	No report	~4702	229,915.23	15.29	4789.90
2750 × 100 × 50 [15,16]	~9365.35	No report	~1665	9126.54	0.14	1250.21
	~56,998.64	0.91	~1814	57,116.37	0.89	1643.16
	~159,597.82	No report	~1383	159,532.14	2.48	1437.53
5000 × 200 × 40 [15]	~2201.04	No report	~502	2207.74	0.02	490.60
	~13,793.67	0.07	~1335	13,840.52	0.07	1274.45
	~38,622.67	No report	~1955	38,744.43	0.20	2094.29

**Table 3:** The actuating and signal read-out methods applicable to different vibration modes of MCL.

Resonant Mode	Actuating Method	Signal Read-Out Method
Bending	piezoelectric, electric, flexoelectric, atomic force, magnetic, ultrasound radiation force and laser irradiation	optics, piezoresistance, piezoelectricity, capacitive, tunneling
Torsional	magnetic	optics, piezoresistance, piezoelectricity

**Table 4:** Max Temperature variation (*K*) in slotted MCL and slotted MCL with edge serrations.

Resonant Mode	Types of Micro Cantilever	Max Temperature Variation (*K*)
1st Bending mode	No serrations	1.796 × 10^−3^
	With serrations	0.481 × 10^−3^
Torsional mode	No serrations	2.928 × 10^−3^
	With serrations	0.263 × 10^−3^

**Table 5:** Viscous and Thermal Dissipation of studied MCL structures.

Resonant Mode	Types of Micro Cantilever	Total Dissipation (W)	Thermal Dissipation (W)	Viscous Dissipation (W)
1st Bending mode	No serrations	1.575 × 10^−8^	8.254 × 10^−13^	1.575 × 10^−8^
	With serrations	0.116 × 10^−8^	0.578 × 10^−13^	0.116 × 10^−8^
Torsional mode	No serrations	4.174 × 10^−9^	4.564 × 10^−12^	4.170 × 10^−9^
	With serrations	0.338 × 10^−8^	0.219 × 10^−12^	0.339 × 10^−9^

## References

[1] Soysal U., Géhin E., Algré E., Berthelot B., Da G., Robine E. (2017). Aerosol mass concentration measurements: Recent advancements of real-time nano/micro systems. J. Aerosol Sci..

[2] Chueinta W., Hopke P.K. (2001). Beta Gauge for Aerosol Mass Measurement. Aerosol Sci. Technol..

[3] Zhu K., Zhang J.J., Lioy P.J. (2007). Evaluation and Comparison of Continuous Fine Particulate Matter Monitors for Measurement of Ambient Aerosols. J. Air Waste Manag. Assoc..

[4] Favez O., Cachier H., Sciare J., Le Moullec Y. (2007). Characterization and contribution to PM 2.5 of semi-volatile aerosols in Paris (France). Atmos. Environ..

[5] Grover B.D. (2005). Measurement of total PM 2.5 mass (nonvolatile plus semivolatile) with the Filter Dynamic Measurement System tapered element oscillating microbalance monitor. J. Geophys. Res..

[6] Jianwen S., Kun Y., Zewen L., Yanwu L. A system of continuous particles monitoring using virtual impactor. Proceedings of the 2015 12th IEEE International Conference on Electronic Measurement & Instruments (ICEMI).

[7] Shendrikar A.D., Steinmetz W.K. (2003). Integrating nephelometer measurements for the airborne fine particulate matter (PM 2.5) mass concentrations. Atmos. Environ..

[8] Hao Z., Abdolvand R., Ayazi F. A High-Q Length-Extensional Bulk-Modemass Sensor with Annexed Sensing Platforms. Proceedings of the 19th IEEE International Conference on Micro Electro Mechanical Systems.

[9] Lee J.E., Zhu Y., Seshia A.A. (2008). A bulk acoustic mode single-crystal silicon microresonator with a high-quality factor. J. Micromech. Microeng..

[10] Natalia N., Gfeller K.Y., Natalia B., Marcel D., Peter L.H., Hans-Joachim G., Martin H. (2007). An antibody-sensitized microfabricated cantilever for the growth detection of Aspergillus niger spores. Microsc. Microanal..

[11] Gupta A., Akin D., Bashir R. (2004). Single virus particle mass detection using microresonators with nanoscale thickness. Appl. Phys. Lett..

[12] Davila A.P., Jang J., Gupta A.K., Walter T., Aronson A., Bashir R. (2007). Microresonator mass sensors for detection of Bacillus anthracis Sterne spores in air and water. Biosens. Bioelectron..

[13] Wasisto H.S., Merzsch S., Waag A., Uhde E., Salthammer T., Peiner E. (2013). Portable cantilever-based airborne nanoparticle detector. Sens. Actuators B Chem..

[14] Wasisto H.S., Merzsch S., Waag A., Uhde E., Salthammer T., Peiner E. (2013). Airborne engineered nanoparticle mass sensor based on a silicon resonant cantilever. Sens. Actuators B Chem..

[15] Wasisto H.S., Merzsch S., Waag A., Uhde E., Salthammer T., Peiner E. (2013). Evaluation of photoresist-based nanoparticle removal method for recycling silicon cantilever mass sensors. Sens. Actuators A Phys..

[16] Wasisto H.S., Uhde E., Peiner E. (2016). Enhanced performance of pocket-sized nanoparticle exposure monitor for healthy indoor environment. Build. Environ..

[17] Dufour I., Josse F., Heinrich S.M., Lucat C., Ayela C., Ménil F., Brand O. (2012). Unconventional uses of microcantilevers as chemical sensors in gas and liquid media. Sens. Actuators B Chem..

[18] Huang M., Yang J., Jun S., Mu S., Lan Y. (2011). Simulation and Analysis of a Metamaterial Sensor Based on a Microring Resonator. Sensors.

[19] Shi L., Xu Y., Tan W., Chen X. (2007). Simulation of optical microfiber loop resonators for ambient refractive index sensing. Sensors.

[20] Kwon H.J., Seok S., Lim G. (2017). System Modeling of a MEMS Vibratory Gyroscope and Integration to Circuit Simulation. Sensors.

[21] Song J., He C., Wang R., Xue C., Zhang W. (2018). A Mathematical Model of a Piezo-Resistive Eight-Beam Three-Axis Accelerometer with Simulation and Experimental Validation. Sensors.

[22] Li J., Tian Y., Dan J., Bi Z., Zheng J., Li B. (2019). Simulation-Based Design and Optimization of Accelerometers Subject to High-Temperature and High-Impact Loads. Sensors.

[23] Feng H., Miao X., Yang Z. (2018). Design, Simulation and Experimental Study of the Linear Magnetic Microactuator. Micromachines.

[24] Sader J.E. (1998). Frequency response of cantilever beams immersed in viscous fluids with applications to the atomic force microscope. J. Appl. Phys..

[25] Van Eysden C.A., Sader J.E. (2006). Resonant frequencies of a rectangular cantilever beam immersed in a fluid. J. Appl. Phys..

[26] Basak S., Raman A., Garimella S.V. (2006). Hydrodynamic loading of microcantilevers vibrating in viscous fluids. J. Appl. Phys..

[27] Van Eysden C.A., Sader J.E. (2009). Frequency response of cantilever beams immersed in compressible fluids with applications to the atomic force microscope. J. Appl. Phys..

[28] Van Eysden C.A., Sader J.E. (2009). Compressible viscous flows generated by oscillating flexible cylinders. Phys. Fluids.

[29] Xia X., Li X. (2008). Resonance-mode effect on microcantilever mass-sensing performance in air. Rev. Sci. Instrum..

[30] Xie H., Vitard J., Haliyo S., Régnier S. (2008). Enhanced sensitivity of mass detection using the first torsional mode of microcantilevers. Meas. Sci. Technol..

[31] Dohn S., Sandberg R., Svendsen W., Boisen A. (2005). Enhanced functionality of cantilever-based mass sensors using higher modes. Appl. Phys. Lett..

[32] Lochon F., Dufour I., Rebière D. (2004). An alternative solution to improve sensitivity of resonant microcantilever chemical sensors: Comparison between using high-order modes and reducing dimensions. Sens. Actuators B Chem..

[33] Qiu H., Xiao D., Feili D., Wu X., Seidel H. (2016). Hydrodynamic analysis of piezoelectric microcantilevers vibrating in viscous compressible gases. Sens. Actuators A Phys..

[34] Venstra W., Westra H., van der Zant H. (2013). Stochastic switching of cantilever motion. Nat. Commun..

[35] Ivaldi P., Abergel J., Matheny M., Villanueva L., Karabalin R., Roukes M., Andreucci P., Hentz S., Defay E. (2011). 50 nm thick AlN film-based piezoelectric cantilevers for gravimetric detection. J. Micromech. Microeng..

[36] Ledermann N., Muralt P., Baborowski J., Forster M., Pellaux J. (2004). Piezoelectric Pb(Zr-x,Ti1-x)O-3 thin film cantilever and bridge acoustic sensors for miniaturized photoacoustic gas detectors. J. Micromech. Microeng..

[37] Keskar G., Elliott B., Gaillard J., Skove M., Rao A. (2008). Using electric actuation and detection of oscillations in microcantilevers for pressure measurements. Sens. Actuators A Phys..

[38] Chen C., Schwarz A., Wiesendanger R., Horn O., Muller J. (2010). Three-electrode self-actuating self-sensing quartz cantilever: Design, analysis, and experimental verification. Rev. Sci. Instrum..

[39] Bhaskar U., Banerjee N.B., Abdollahi A., Wang Z., Schlom D., Rijnders G., Catalan G. (2016). A flexoelectric microelectromechanical system on silicon. Nat. Nanotechnol..

[40] Schwarz K., Rabe U., Hirsekorn S., Arnold W. (2008). Excitation of atomic force microscope cantilever vibrations by a Schottky barrier. Appl. Phys. Lett..

[41] Penedo M., Raman A.R., Hormeno S.H., Fernandez-Martinez I., Luna M., Briones F. (2014). Enhanced efficiency in the excitation of higher modes for atomic force microscopy and mechanical sensors operated in liquids. Appl. Phys. Lett..

[42] Huber T., Abell B., Mellema D., Spletzer M., Raman A. (2010). Mode-selective noncontact excitation of microcantilevers and microcantilever arrays in air using the ultrasound radiation force. Appl. Phys. Lett..

[43] Marti O., Ruf A., Hipp M., Bielefeldt H., Colchero J., Mlynek J. (1992). Mechanical and thermal effects of laser irradiation on force microscope cantilevers. Ultramicroscopy.

[44] Hoummady M., Farnault E., Yahiro T., Kawakatsu H. (1997). Simultaneous optical detection techniques, interferometry, and optical beam deflection for dynamic mode control of scanning force microscopy. J. Vac. Sci. Technol. B.

[45] Rugar D., Mamin H.J., Guethner P. (1989). Improved Fiber-Optic Interferometer for Atomic Force Microscopy. Appl. Phys. Lett..

[46] Thaysen J., Boisen A., Hansen O., Bouwstra S. (2000). Atomic force microscopy probe with piezoresistive read-out and a highly symmetrical Wheatstone bridge arrangement. Sens. Actuators A Phys..

[47] Linnemann R., Gotszalk T., Hadjiiski L., Rangleow I. (1995). Characterization of a Cantilever with an Integrated Deflection Sensor. Thin Solid Films.

[48] Shin S., Kim J., Sim S., Lee J. (2008). A multisized piezoelectric microcantilever biosensor array for the quantitative analysis of mass and surface stress. Appl. Phys. Let..

[49] Verd J., Abadal G., Teva J., Gaudo M., Uranga A., Borrise X., Campabadal F., Esteve J., Costa E., Perez-Murano F. (2005). Design, fabrication, and characterization of a submicroelectromechanical resonator with monolithically integrated CMOS readout circuit. J. Microelectromech. Syst..

[50] Kenny T., Kaiser W., Podosek J., Rockstad H., Reynolds J., Vote E. (1993). Micromachined Tunneling Displacement Transducers for Physical Sensors. J. Vac. Sci. Technol. A Vac. Surfaces Films.

[51] Rabe U., Janser K., Arnold W. (1996). Vibrations of free and surface-coupled atomic force microscope cantilevers: Theory and experiment. Rev. Sci. Instrum..

[52] Van Eysden C., Sader J. (2008). Frequency response of cantilever beams immersed in viscous fluids with applications to the atomic force microscope: Arbitrary mode order. J. Appl. Phys..

[53] Coppola G., Capuano F., de Luca L. (2019). Discrete Energy-Conservation Properties in the Numerical Simulation of the Navier-Stokes Equations. Appl. Mech. Rev..

[54] Ikeda T., Ueda T., Nakata T., Noda R., Tanaka H., Fujii T., Liu H. (2018). Morphology Effects of Leading-edge Serrations on Aerodynamic Force Production: An Integrated Study Using PIV and Force Measurements. J. Bionic Eng..

[55] Rao C., Ikeda T., Nakata T., Liu H. (2017). Owl-inspired leading-edge serrations play a crucial role in aerodynamic force production and sound suppression. Bioinspir. Biomim..

[56] Bednorz A. (2006). Sutherland formula for a square-well fluid. Phys. Rev. E.

[57] Akaogi M., William M.W. (2017). Clapeyron’s Equation. Encyclopedia of Geochemistry.

[58] Blackstock D.T. (2001). Fundamentals of Physical Acoustics. J. Acoust. Soc. Am..

